# Anti-depressant effects of ethanol extract from *Cannabis sativa* (hemp) seed in chlorpromazine-induced *Drosophila melanogaster* depression model

**DOI:** 10.1080/13880209.2021.1949356

**Published:** 2021-08-06

**Authors:** Yejin Ahn, Sung Hee Han, Min Guk Kim, Ki-Bae Hong, Woo Jung Kim, Hyung Joo Suh, Kyungae Jo

**Affiliations:** a Department of Integrated Biomedical and Life Science, Graduate School, Korea University, Seoul, Republic of Korea; b Institute of Human Behavior & Genetic, College of Medicine, Korea University, Seoul, Republic of Korea; c Department of Food Science and Nutrition, Jeju National University, Jeju, Republic of Korea; d Biocenter, Gyeonggido Business and Science Accerlerator, Suwon, Republic of Korea

**Keywords:** Behavioural pattern, cannabinoid, neurotransmitter

## Abstract

**Context:**

Depression is a severe mental illness caused by a deficiency of dopamine and serotonin. *Cannabis sativa* L. (Cannabaceae) has long been used to treat pain, nausea, and depression.

**Objective:**

This study investigates the anti-depressant effects of *C. sativa* (hemp) seed ethanol extract (HE) in chlorpromazine (CPZ)-induced *Drosophila melanogaster* depression model.

**Materials and methods:**

The normal group was untreated, and the control group was treated with CPZ (0.1% of media) for 7 days. The experimental groups were treated with a single HE treatment (0.5, 1.0, and 1.5% of media) and a mixture of 0.1% CPZ and HE for 7 days. The locomotor activity, behavioural patterns, depression-related gene expression, and neurotransmitters level of flies were investigated.

**Results:**

The behavioural patterns of individual flies were significantly reduced with 0.1% CPZ treatment. In contrast, combination treatment of 1.5% HE and 0.1% CPZ significantly increased subjective daytime activity (*p* < 0.001) and behavioural factors (*p* < 0.001). These results correlate with increased transcript levels of dopamine (*p* < 0.001) and serotonin (*p* < 0.05) receptors and concentration of dopamine (*p* < 0.05), levodopa (*p* < 0.001), 5-HTP (*p* < 0.05), and serotonin (*p* < 0.001) compared to those in the control group.

**Discussion and conclusions:**

Collectively, HE administration alleviates depression-like symptoms by modulating the circadian rhythm-related behaviours, transcript levels of neurotransmitter receptors, and neurotransmitter levels in the CPZ-induced *Drosophila* model. However, additional research is needed to investigate the role of HE administration in behavioural patterns, reduction of the neurotransmitter, and signalling pathways of depression in a vertebrate model system.

## Introduction

Depression is one of the most prevalent mental disorders and causes significant difficulties and disabilities, affecting people’s thoughts, emotions, behaviour, physical functioning, and overall quality of life (Keles et al. 2020). Symptoms of depression include fatigue, concentration difficulties, sleep disorder, and changes in weight or appetite. The major causes of depression reported are functional imbalances and deficiencies of monoamine-based neurotransmitters, including dopamine, serotonin, and norepinephrine (Dean and Keshavan 2017; Brennenstuhl et al. 2019). The regulation of dopamine levels in the prefrontal cortex is a target of many pharmacological treatments and natural substances in the treatment of depression. The prefrontal cortex is regulated by the neurotransmission of catecholamines and incorporates cognitive and emotional information. Upregulation of dopamine receptor D1 due to low levels of dopamine in the prefrontal cortex is a remarkable factor for depression (Lavergne and Jay 2010). Several studies have demonstrated that dopamine metabolites and receptors in both cerebrospinal fluid and brain regions affect mood and motivation (Dunlop and Nemeroff 2007).

Anti-depressants typically work by blocking the reuptake of certain neurotransmitters (norepinephrine, serotonin, and dopamine) to the post-synaptic neuron. Anti-depressants that are mainly used are monoamine oxidase inhibitors which improve the function of monoamine transporters, and tricyclics that increase the levels of norepinephrine and serotonin (Thanacoody 2020). Recently, selective serotonin reuptake inhibitors, such as fluoxetine (Prozac), which selectively act on the serotonin system, are being used (Kumar and Sharma 2020). However, these medications have shown negative side effects, such as sexual dysfunction, vomiting, diarrhoea, constipation, gastrointestinal disorders, loss of appetite, dry mouth, anxiety, and insomnia (Ries et al. 2017). Therefore, many people are seeking naturally derived anti-depressants.

Several natural products, such as *Hypericum perforatum* L. (Hypericaceae), *Rhodiola rosea* L. (Crassulaceae)*, Vitis vinifera* L. (Vitaceae), and *Bupleurum falcatum* L. (Apiaceae), have been known to be effective in depression (Nathan 2001; Kurkin et al. 2006; Kwon et al. 2010; Xu et al. 2010). Further, polyphenols (such as chlorogenic acid, curcumin, resveratrol, and proanthocyanidins), flavonoids (such as rutin, tannin, and quercetin), and cannabinoids (such as tetrahydrocannabinol, cannabichromene, and cannabidiol) are known to alleviate depression (Noldner and Schotz 2002; Anjaneyulu et al. 2003; Kulkarni et al. 2008; El-Alfy et al. 2010; Xu et al. 2010; Pathak et al. 2013; Chandrasekhar et al. 2017; Zhu et al. 2019).

Previous studies have shown that depression can be regulated by *Cannabis sativa* L. (Cannabaceae), which contains compounds, such as antidepressants and anxiolytics, suggesting that *C. sativa* extract is associated with cellular and molecular changes in brain regions (de Mello Schier et al. 2014; Silote et al. 2019). *C. sativa* has long been recognized and valued as an important source of food, fibre, and medicine throughout Asia, India, and Russia since ancient times (Russo 2007). Cannabidiol (CBD) is a major component of *C. sativa* and has anxiolytic, antipsychotic, neuroprotective, and anti-depressant effects (Campos et al. 2016; Ligresti et al. 2016). CBD reduces immobility time in a forced swim test (FST) of a trained helplessness mouse model and decreases brain-derived neurotrophic factor levels in the hippocampus and frontal cortex to a level similar to that in imipramine (a positive control)-treated mouse (Reus et al. 2011). In addition, CBD has been found to induce anti-psychotic and anti-anxiety effects in preclinical and clinical studies (Zuardi et al. 1982, 2006; Resstel et al. 2006), improve immune regulation, and cognitive function, and show anti-inflammatory and neuroprotective effects (Weston-Green 2018).

In this study, hemp seed ethanol extract (HE) containing CBD was used to evaluate its anti-depressant effect and mechanism of action through gene expression of receptors in an invertebrate model. *Drosophila melanogaster* is a useful invertebrate model for the discovery and development of early-stage synthetic chemistry and natural products for human neurodegenerative disorders, including Alzheimer's, Huntington, and Parkinson's disease (PD) (Lee and Min 2015; Hood and Amir 2017; Bolus et al. 2020). In *Drosophila* and humans, several neurobiological processes are similar because they use the same neurotransmitter and have similar neuronal signalling mechanisms (O'Kane 2011; Bellen et al. 2010). PD is a movement disorder and is characterized by loss of dopaminergic neurons, and *Drosophila*'s dopamine synthesis pathway is human-like (Aryal and Lee 2019; Chia et al. 2020). Neurotoxin-treated flies induced a PD phenotype, including decreased dopaminergic neurons and motor activity, and increased oxidative stress (Shukla et al. 2014; Cassar et al. 2015). Therefore, *Drosophila* is an effective model for studying human diseases and screening potential therapeutic drugs, including neurological disorders, such as sleep disorders and depression.

Recent studies suggest that *Drosophila* has caused a depression-like state under chronic mild stress, vibration stress, and drug treatment. (Jiang et al. 2017; Ries et al. 2017; Araujo et al. 2021). Unpredictable chronic mild stress (UCMS) in *Drosophila* induced behavioural changes (anhedonia, aggression, immobility, reduction mating) due to decrease of monoamine levels. However, administration of γ-oryzanol from rice bran oil relieves symptoms of depression in fruit flies caused by UCMS through changes in molecular and genetic factors (Ries et al. 2017). Repeated vibration-stress in *Drosophila* also reduced voluntary behavioural activity due to a lack of serotonergic neuron signals. (Araujo et al. 2021). In addition, exposure to drugs, such as levodopa (L-DOPA) or chlorpromazine (CPZ) induces a depression-like phenotype in adult fruit flies, including decreased appetite, sexual activity, and serotonin levels. Major biochemical markers associated with lipid peroxidation and oxidative stress are also observed to change. Assessed using RNA-sequencing and quantitative real-time polymerase chain reaction (qRT-PCR) experiments, changes in gene expression associated with metabolic and neurological disorders after CPZ exposure were reported (Jiang et al. 2017). Thus, the anti-depressant effect of HE was confirmed by the CPZ-induced depression *Drosophila* model. HE was extracted using 70% ethanol, and cannabinoid derivatives were analyzed by high-performance liquid chromatography (HPLC). Additionally, the anti-depressant effects of HE were evaluated by measuring the locomotor activity, neurotransmitter-related mRNA expression, and depression-related neurotransmitter levels using a CPZ-induced *D. melanogaster* model.

## Materials and methods

### Materials

*Cannabis sativa* seeds were purchased the Organica (Chungju, Republic of Korea) in 2019, and the sample was identified by Professor Shin, Department of Food Science and Biotechnology, Kyonggi University, South Korea. Voucher specimens (*Cannabis sativa* L.: FSB-2019-04) were deposited at the same department. For HPLC analysis, all solvents used were HPLC-grade and purchased from Fisher Scientific (Cleveland, OH, USA). The cannabinoids cannabidivarin (CBDV), cannabidiolic acid (CBDA), cannabidivarinic acid (CBDVA), tetrahydrocannabivarin (THCV), and Δ9-tetrahydrocannabinolic acid-A (THCA-A) were purchased from Sigma-Aldrich (St. Louis, MO, USA). All other chemicals and reagents were of the highest grade available.

### Extraction of HE

Ethanol (70%) was added to hemp seed in a ratio of 1:10 (w/v) and extracted three times at 25 °C for 24 h. Then, the extract was passed through a filter paper (6.5 cm discs of Whatman No. 1) and evaporated at 35 °C using a rotary vacuum evaporator (V-100; Büchi, Labortechnik, Germany). The ethanol extract was lyophilized and stored at −20 °C for further experiments.

### *Drosophlia* stocks

Wild-type *Drosophila* (Canton-S strain) were obtained from the Bloomington *Drosophila* Stock Centre at Indiana University. The flies were raised on standard medium (sucrose, cornmeal, dried yeast, agar, propionic acid, and *p*-hydroxybenzoic acid methyl ester solution) at 25 ± 1 °C with 60% relative humidity and a 12 h light: dark cycle. Before treatment, each sample of 3-day-old male flies was collected under CO_2_ anaesthesia. All *Drosophila* were cultured with sucrose-agar media (5% sucrose and 2% agar) for the experiment. CPZ and HE were dissolved in distilled water and mixed with a sucrose-agar medium. CPZ (0.1% of medium) was used to induce depression in *Drosophila*. In all experiments, the normal group was untreated, and the control group was treated with 0.1% CPZ. In the locomotor activity assay, 0.5, 1.0, and 1.5% HE were mixed with 0.1% CPZ to analyze the effect of HE according to the concentration. In behavioural tests, gene expression analysis, and HPLC assay, various concentrations of single HE treatment (0.5, 1.0, and 1.5%) and a mixture of 0.1% CPZ and 1.5% HE were used to evaluate the antidepressant effect of HE.

### Cannabinoids and depression-related neurotransmitters analysis

CBDV, CBDA, CBDVA, THCV, and THCA-A were dissolved in methanol and then determined using a previously described HPLC method (Mandrioli et al. 2019). Six-point standard calibration curves for five cannabinoids were prepared at concentrations ranging from 6.25 to 200 μg/mL. Each cannabinoid in HE was measured using a Water Alliance HPLC system (Waters, Milford, MA, USA) equipped with a Meteoric Core C18 BIO column (4.6 × 150 mm, 2.7 μm; YMC, Kyoto, Japan) and a photodiode detector. The mobile phase was 91% acetonitrile with 0.05% trifluoroacetic acid, and the flow rate was 0.5 mL/min. The detect wavelength of cannabinoids was 217 nm.

Analysis of depression-related neurotransmitters levels was performed by HPLC-fluorescence detection (FD) according to a previously reported method (Denno et al. 2015). All the experiments were performed in triplicate (50 flies per replicate). Then, the head of the flies was removed from the body and homogenized in 200 μL phosphate-buffered saline (pH 7.4). The homogenate was ultrasonicated for 15 min and then centrifuged at 12,000 × *g* for 15 min at 4 °C. The supernatant was left on ice for 5 min and then analyzed by HPLC-FD. Sample aliquots (20 μL) were injected into a Waters Alliance e2695 HPLC system (Waters) equipped with a Waters 2475 Multi-λ fluorescence detector. The analytical column was a YMC-Pack Pro C18 column (250 × 4.6 mm, 5 μm; YMC), and the temperature was controlled at 35 °C. Detection was performed at excitation and emission wavelengths of 270 and 320 nm, respectively. For the analysis of epinephrine, dopamine, L-DOPA, the mobile phase was acetate buffer (pH 4.0, 12 mM acetic acid, 0.26 mM Na_2_EDTA)-methanol (86:14, v/v) and run at a flow rate of 1.0 mL/min for 20 min. In addition, analysis of 5-hydroxytryptophan (5-HTP) and serotonin was performed using a mobile phase of acetate buffer (pH 4.0, 12 mM acetic acid, 0.26 mM Na_2_EDTA)-methanol (90:10, v/v) and run at a flow rate of 0.5 mL/min for 60 min. The concentration of the prepared standard solutions ranged from 12.5 to 200 ng/mL.

### Locomotor activity assay

For the *Drosophila* activity monitoring (DAM) system (TriKinetics, Waltham, MA, USA), that is for analysis of behaviour patterns, flies were kept in individual glass tubes and subjected to a 24 h adaptation period. All recordings were performed for 7 d under constant darkness at 24 ± 1 °C. The experiments were performed in triplicate (10 flies per replicate). Data were recorded using the DAM management software (TriKinetics) with controls for environmental stimuli, such as sound and light. The number of infra-red detector interruptions at each time interval was recorded and visualized using Actogram J software (NIH, Bethesda, MD, USA). The actograms were used to visualize circadian rhythm by measuring the total activity divided by subjective daytime and night-time. Subjective daytime and night-time indicate the day (10:00–22:00) and night (22:00–10:00) phases, respectively. The locomotor activity was recorded at 1 min intervals to measure behaviour, and the subjective daytime and night-time activities were the sums of the total number of movements observed during the day or night periods, respectively. The total sleep time was calculated as the sum of the total sleep during the subjective light and dark phases. Sleep was defined as a period of uninterrupted behavioural immobility and inactivity lasting more than 5 min (0 counts per min) (Hendricks et al. 2000). In addition, the number of sleep episodes was counted and summed up (Ko et al. 2016).

### Behavioural tests

The open-field test (OFT) was performed using a video-tracking system according to a previously reported method (Kaur et al. 2015). For the video-tracking system, the flies allowed to acclimate to the chamber for 1 min, and 30 flies per group were used. This was followed by a 5 min observation period during which their activities were recorded on the video track of a tape. The chamber consisted of a circular arena (8 mm in diameter and 0.1 mm in height), with a white background. Multiple parameters, including the total distance moved, velocity, the number of episodes of activity and inactivity, and mobility were assessed for the characterization of locomotor activity using the Noldus EthoVision-XT system (Noldus Information Technology, Netherlands). The distance moved was used as an estimate of the general activity of flies, and the velocity of the movement was measured as the walking speed of flies. *D. melanogaster* movements were divided into ‘moving’ and ‘not moving’, and spatial displacement thresholds were applied as a marker of activity (centre point moving) and inactivity (centre point not moving) according to a previously described method (Martin and Krantz 2014). ‘Moving’ was defined as movement over the speed of 0.1 cm/s, while ‘not moving’ was defined as movement below 0.05 cm/s. Mobility was defined based on changes in the pixels of the sample and calculated independent of movement of the centre point (Kaur et al. 2015).

### qRT-PCR

The experiment was performed in a 12 h light/dark cycle for 7 d (3 d: adaptation and 7 d: experiment). Total RNA was extracted from heads of 10-day-old flies using TRIzol reagent (Invitrogen, CA, USA), while genomic DNA was isolated using RQ1 RNase-free DNase I (Promega, WI, USA) according to the manufacturer’s protocol. All the experiments were performed in triplicate (50 flies per replicate). The quality of RNA samples was determined by high optical density ratios (A260/A280 > 1.8), and 1 μg of total RNA was reverse transcribed using SuperScript III Reverse Transcriptase (Invitrogen) and oligo d(T) as the primer. qRT-PCR was performed on the resulting cDNA using a Power Taqman PCR Master Mix kit (Applied Biosystems, CA, USA). For real-time PCR, the cycle conditions were 50 °C for 2 min, 95 °C for 10 min, followed by 50 cycles of 95 °C for 15 s and 60 °C for 1 min. Quantitative analyses were conducted using StepOne plus Software V. 2.0 (Applied Biosystems), and results were normalized to a validated control gene, *RpL32* (NM_001144655.3), using the ΔΔC_T_ method (Livak and Schmittgen 2001). The target genes examined using qRT-PCR were as follows: Dopamine 1-like receptor 1 (*Dop1R1*, NM_001170136.2) and 5-hydroxytryptamine receptor 1 A (*5-HT1A*, NM_166322.2).

### Statistical analysis

All statistical analyses were performed using the Statistical Package for Social Sciences version 12.0 (SPSS Inc., Chicago, IL, USA). Differences between groups were evaluated using a one-way analysis of variance and Tukey's multiple comparison test at a level of significance of *p* < 0.05. Different symbols indicate significant differences at ^#^*p* < 0.05, ^##^*p* < 0.01, and ^###^*p* < 0.001 *vs.* normal group. Additionally, *p < 0.05, **p < 0.01, and ***p < 0.001 *vs.* control group. All data are reported as mean ± standard error of the mean (SEM).

## Results

### Cannabinoids analysis in HE

**Figure 1.**
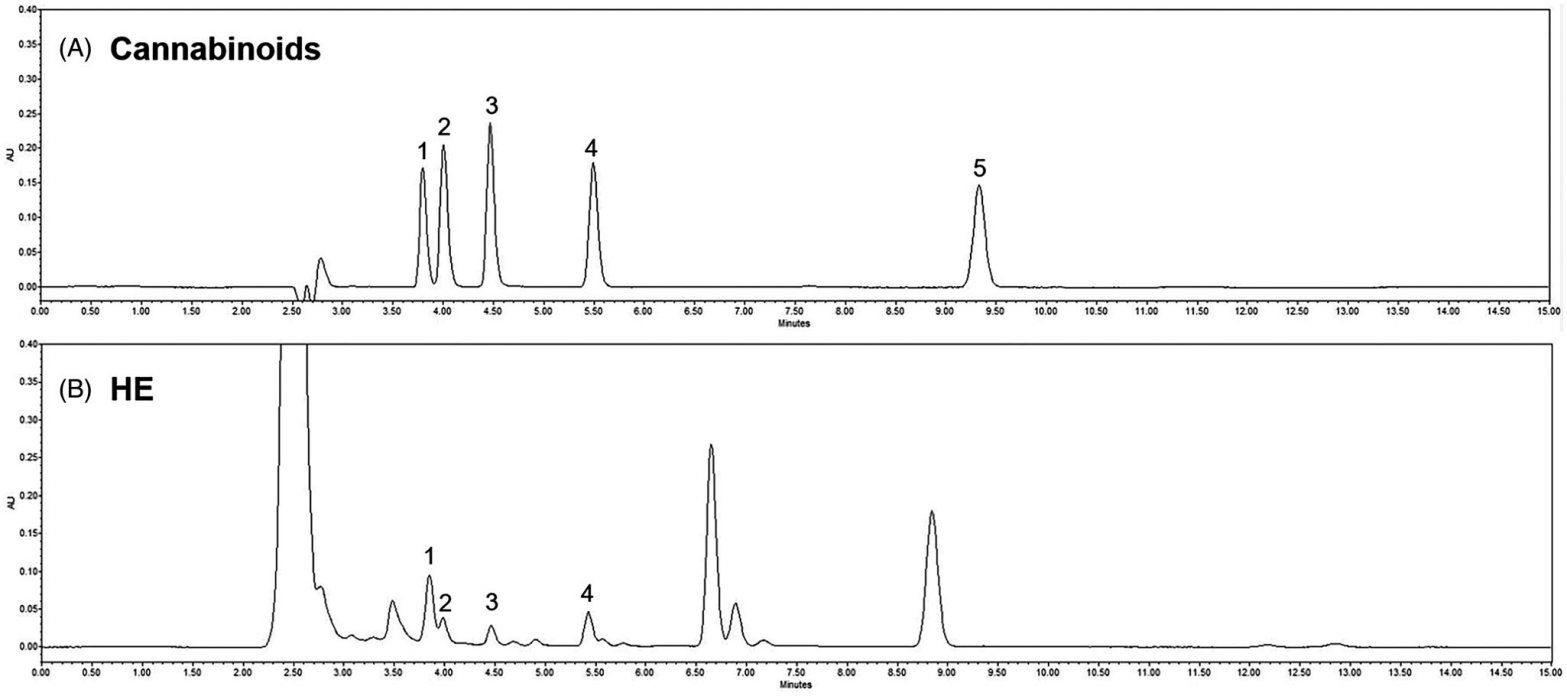
High performance liquid chromatography for cannabinoids in hemp seed ethanol extract (HE). (A) Standard cannabinoids and (B) cannabinoids in HE. 1: cannabidivarinic acid; 2: cannabidivarin; 3: cannabidiolic acid; 4: tetrahydrocannabivarin; and 5: tetrahydrocannabinolic acid-A.

The active compounds in hemp seed extract are known as cannabinoids. Analysis of cannabinoids in HE was confirmed by HPLC. In Figure 1, peaks were identified by comparing the retention time of HE to that of a standard solution. Figure 1 shows that HE contains CBDVA, CBDV, CBDA, and THCV among the various cannabinoids. In addition, a high-resolution mass spectrometer (HR-MS) was performed for accurate identification of compounds. Mass fragmentation spectra in positive mode are reported in Supplementary Figure 1. All cannabinoids in HE were identified according to their chemical formula, retention time, molecular ion [M + H]^+^, and mass accuracy (Δppm) (Supplementary Table 1).

### Effects of HE on locomotor activity

**Figure 2.**
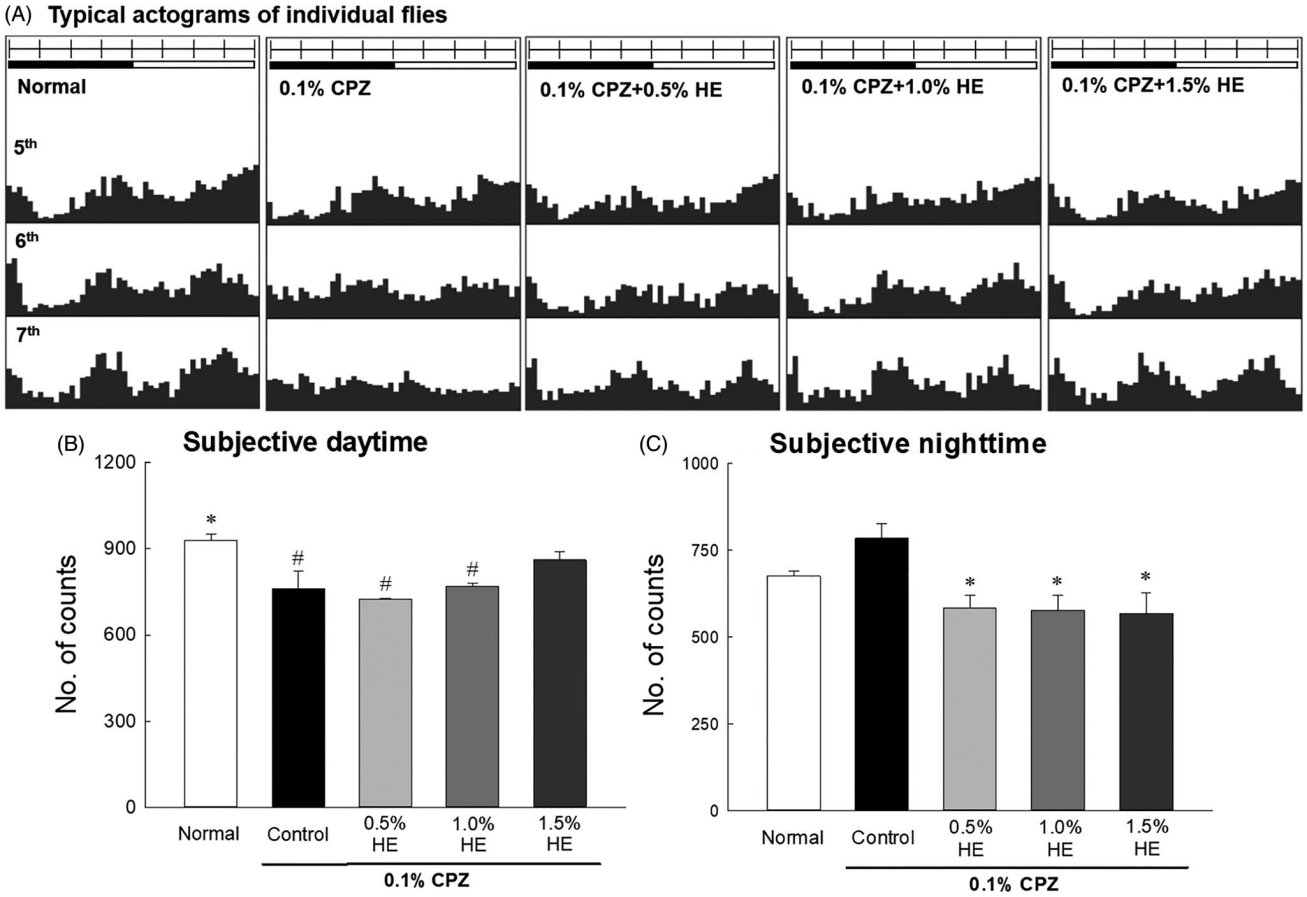
Effects of hemp seed ethanol extract (HE) on the locomotor activity of CPZ-induced *Drosophila melanogaster* depression model. This experiment was performed under constant darkness for 7 days. Average activity in a 30 min interval was calculated over 7 days. (A) Typical actograms of individual normal flies, control flies exposed to 0.1% CPZ, and flies exposed to different doses of HE and 0.1% CPZ. Locomotor activity during subjective (B) daytime and (C) night-time assessed using the *Drosophila* Activity Monitor system. Black and white bars on top of the actograms indicate dark and light, and the dark was 22:00–10:00 and the light phase was 10:00–22:00, respectively. Data are expressed as mean ± standard error of the mean (SEM) for each group (*n* = 10). Different symbols indicate significant differences at ^#^*p* < 0.05 *vs.* normal group, and *p < 0.05 *vs.* control group. CPZ: chlorpromazine.

The effects of HE were investigated by measuring the locomotor activities of CPZ-treated flies using the DAM system. The results revealed that 0.1% CPZ decreased movement during the subjective daytime and disrupted the circadian rhythm of the flies; the circadian rhythms were irregular in frequency (vertical axis) and amplitude (horizontal axis) (Figure 2(A)). The CPZ-induced reduction in activity increased most effectively on the 7th day of HE administration (Figure 2(A)). Figures 2(B,C) showed that the locomotor activity was combined and calculated over 7 d. The activity of 0.1% CPZ-treated flies in the control group significantly decreased during the subjective daytime compared to the normal group (Figure 2(B); 0.82-fold; *p* < 0.035). The activity of the control group seemed to increase during subjective night-time compared to the normal group, and there was no significant difference (Figure 2(C)). Flies administered the combination of HE and CPZ had reduced activity during the subjective night-time compared to the control group. All concentrations of HE (0.5, 1.0, and 1.5%) with CPZ decreased subjective night-time activity in flies compared to the control (Figure 2(C); *p* < 0.050).

**Figure 3.**
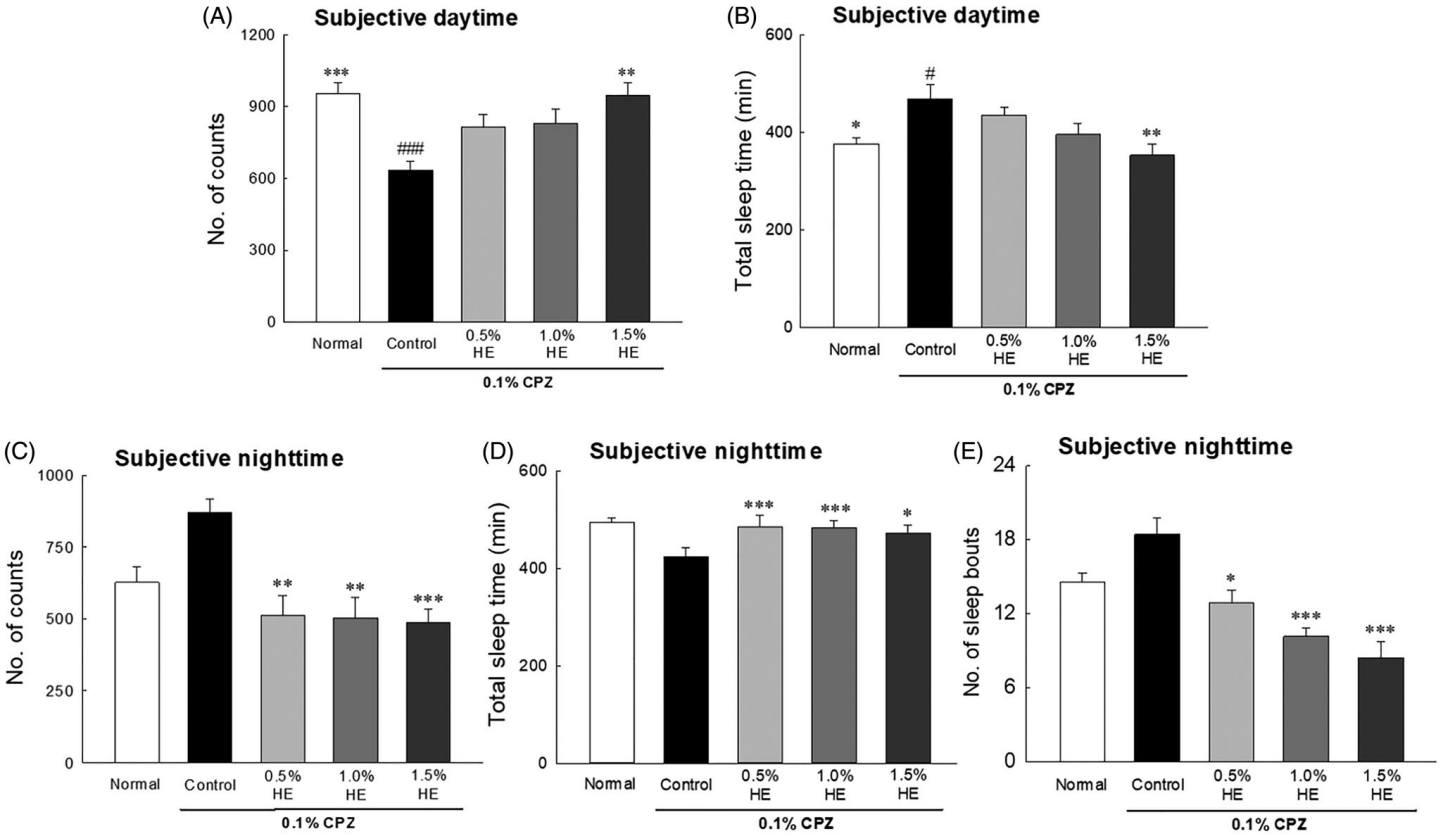
Effects of hemp seed ethanol extracts (HE) on behavioural patterns in CPZ-induced *Drosophila melanogaster* depression model. Locomotor activity in a 30 min interval was calculated at 7 days. Subjective daytime (A) activity and (B) total amount of sleep; subjective night-time (C) activity and (D) total amount of sleep; and (E) number of sleep bouts of normal group, control group and treatment groups using the *Drosophila* Activity Monitor (DAM) system. Data are expressed as mean ± standard error of the mean (SEM) for each group. Different symbols indicate significant differences at ^#^*p* < 0.05, and ^###^*p* < 0.001 *vs.* normal group, and *p < 0.05, **p < 0.01, and ***p < 0.001 *vs.* control group. CPZ: chlorpromazine.

The effects of HE on the locomotor activities of CPZ-induced flies on the 7th day are shown in Figure 3. The flies in the control group had significantly reduced locomotor activities during the subjective daytime compared to normal (Figure 3(A); 0.67-fold; *p* < 0.001), and significantly increased total sleep time of the subjective daytime compared to normal (Figure 3(B); 1.25-fold; *p* < 0.035). Flies administered with 1.5% HE and CPZ showed significantly different subjective daytime (Figure 3(A); *p* < 0.002) and night-time activity (Figure 3(C); *p* < 0.001) compared to those in the control group. In the CPZ-induced depression model, when 1.5% HE was administered, locomotor activity during the subjective daytime significantly increased by 1.49-fold compared to control (Figure 3(A); *p* < 0.002). In addition, during the subjective night-time, 1.5% HE administration significantly decreased the locomotor activity and number of sleep episodes by 0.56- and 0.46-fold, respectively, compared to control (Figures 3(C,E); *p* < 0.001).

### Effects of ethanol extract from HE on behavioural patterns

**Figure 4.**
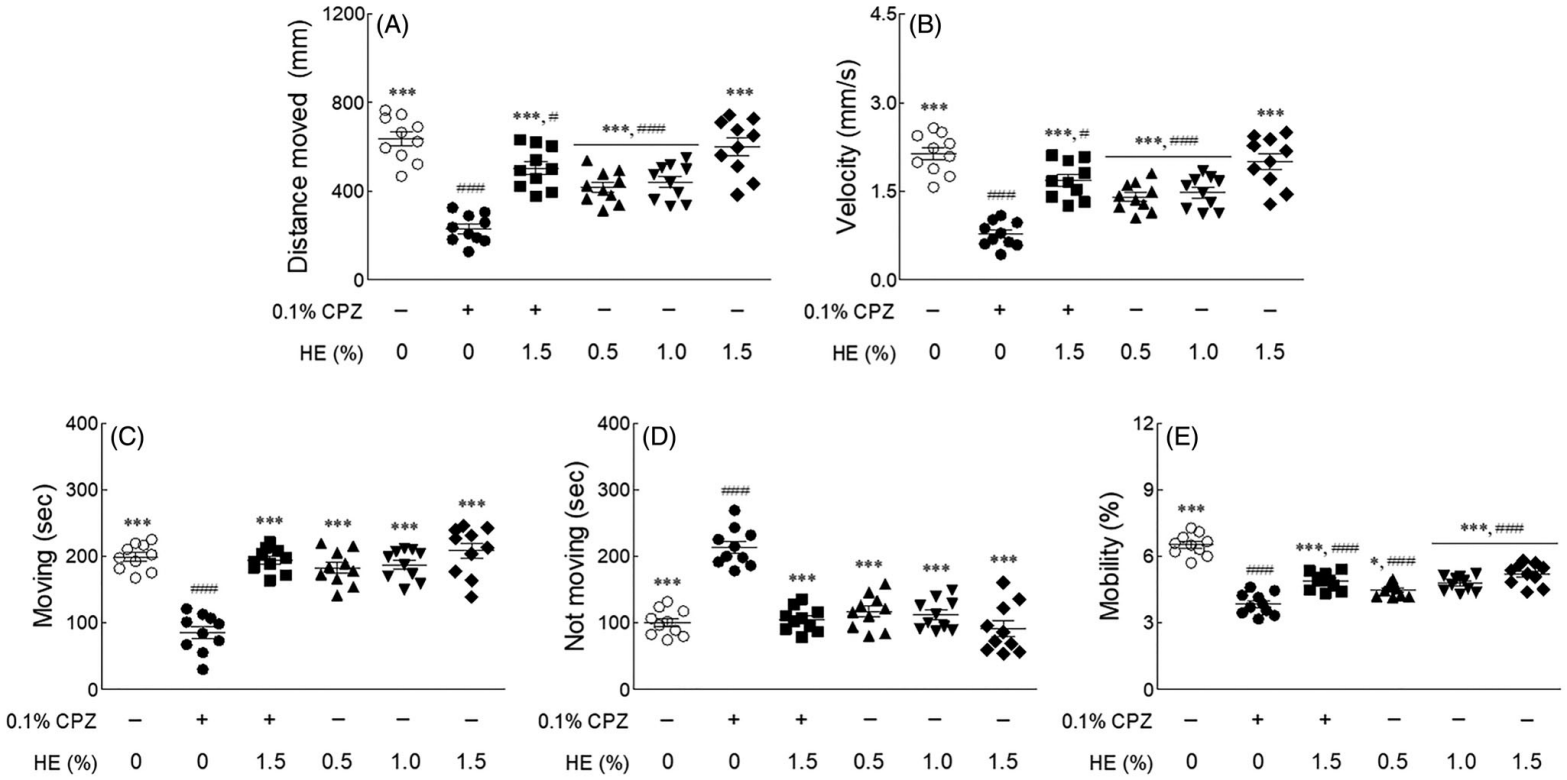
Effects of hemp seed ethanol extract (HE) on locomotor ability in CPZ-induced *Drosophila melanogaster* depression model. (A) Distance moved, (B) velocity, (C) moving, (D) not moving, and (E) mobility of normal group, control group and treatment groups using the open field test. After 7 days of HE and/or chlorpromazine administration, the locomotor activities during the 5 min observation period in the video tracking were analyzed using EthoVision-XT system. Data are expressed as mean ± standard error of the mean (SEM) for each group. Different symbols indicate significant differences at ^#^*p* < 0.05, and ^###^*p* < 0.001 *vs.* normal group, and *p < 0.05, and ***p < 0.001 *vs.* control group. CPZ: chlorpromazine.

The effect of HE on behavioural changes in fruit flies was measured using the Noldus EthoVision-XT system. This assay measured several factors, such as distance moved, velocity, movement (moving and not moving), and mobility during the subjective daytime period. As shown in Figures 4(A–C,E), the distance moved (Figure 4(A); *p* < 0.001), velocity (Figure 4(B); *p* < 0.001), moving (Figure 4(C); *p* < 0.001), and mobility (Figure 4(E); *p* < 0.001) were significantly reduced in the 0.1% CPZ-treated control group compared to the normal group. In contrast, the inactivity (centre point not moving) of flies in the control group was significantly increased compared to that of flies in the normal group (Figure 4(D); *p* < 0.001). In only 1.5% HE-treated group, the distance moved, velocity, moving, and immobility time was similar to those in the normal group. However, administration of 1.5% HE and CPZ significantly increased the distance moved (2.19-fold), velocity (2.19-fold), moving (2.28-fold), and mobility (1.27-fold) compared to the control group (Figures 4(A–C,E); *p* < 0.001). Additionally, the inactivity of flies (0.49-fold) significantly decreased compared to that in the control group (Figure 4(D); *p* < 0.001). In particular, the movements (moving and not moving) of flies treated with 1.5% HE and CPZ were similar to those in the normal group (Figures 4(C,D)).

### Effects of HE on mRNA expression

**Figure 5.**
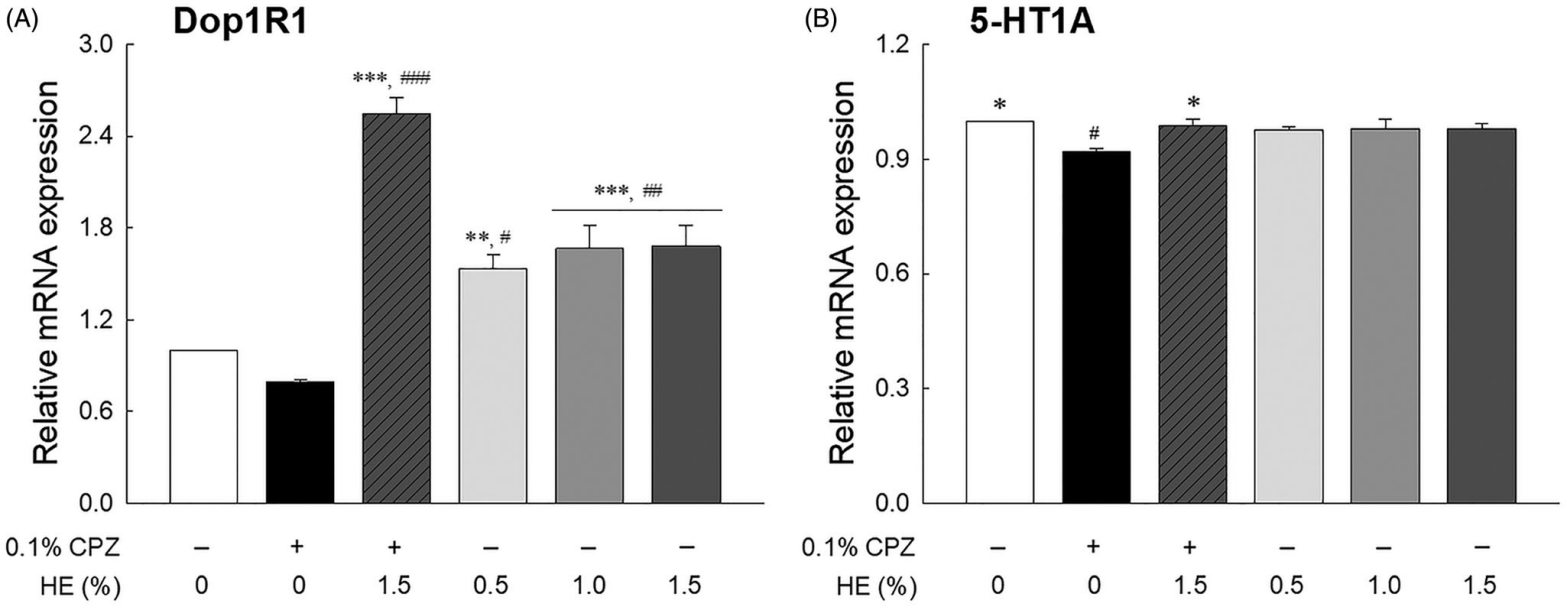
Effects of hemp seed ethanol extract (HE) on (A) *Dop1R1* and (B) *5-HT1A* mRNA expression in *Drosophila melanogaster* heads. After 7 d of HE and/or CPZ administration, the head of *D. melanogaster* was homogenized, and the expression levels of genes in the homogenates were estimated. Data are expressed as mean ± standard error of the mean (SEM) for each group. Different symbols indicate significant differences at ^#^*p* < 0.05, ^##^*p* < 0.01, and ^###^*p* < 0.001 *vs.* normal group, and *p < 0.05, **p < 0.01, and ***p < 0.001 *vs.* control group. CPZ: chlorpromazine; *Dop1R1*: dopamine 1-like receptor 1; *5-HT1A*: 5-hydroxytryptamine receptor.

The effects of HE on the mRNA expression of *Dop1R1* and *5-HT1A* (serotonin receptor) are shown in Figure 5. Although not statistically significant, transcript levels of *Dop1R1* were seemed to be lower in the CPZ-treated control group than the normal group (Figure 5(A); *p* < 0.720). The transcript levels of *5-HT1A* in the control group were significantly lower than that those of the normal group (Figure 5(B); *p* < 0.045). The *Dop1R1* transcript levels in the HE treatment groups (0.5, 1.0, and 1.5%) significantly increased compared to those in the normal group (*p* < 0.030 and *p* < 0.006, respectively). In contrast, the *5-HT1A* levels in the HE treatment groups were similar to those in the normal group. Further, the flies administered 1.5% HE and CPZ had significantly increased transcript levels of *Dop1R1* (3.19-fold; *p* < 0.001) and *5-HT1A* (1.08-fold; *p* < 0.045) compared to those in the control group (Figure 5). In particular, the *Dop1R1* transcript levels in the 1.5% HE and CPZ-treated group were higher than those in the 1.5% HE-treated group, despite CPZ treatment.

### Effects of HE on depression-related neurotransmitters levels

**Figure 6.**
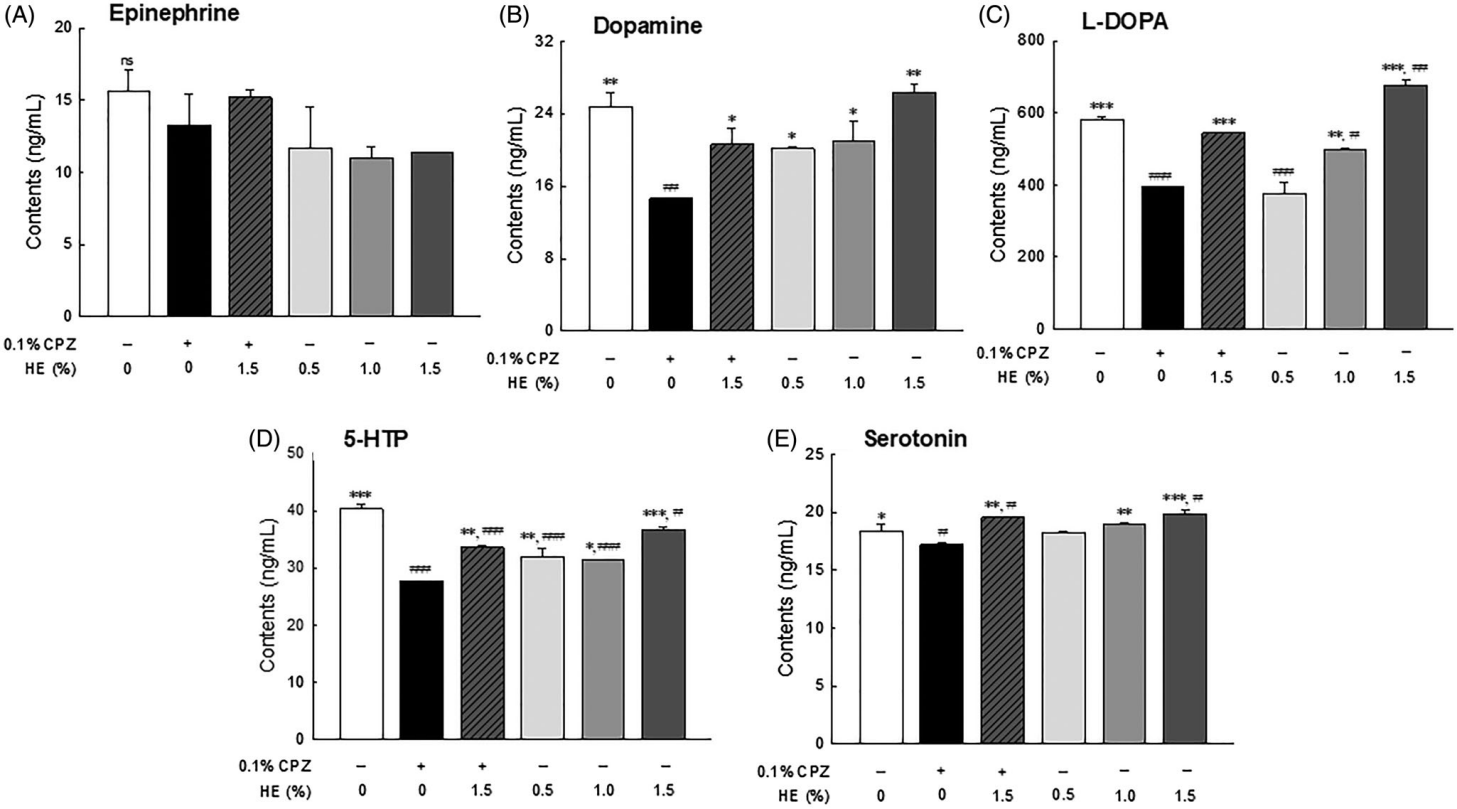
Effects of hemp seed ethanol extract (HE) on levels of depression-related neurotransmitters (epinephrine, dopamine, L-DOPA, 5-HTP, and serotonin) in *Drosophila melanogaster* heads. After 7 days of HE and/or CPZ administration, the head of *D. melanogaster* was homogenized, and the levels of catecholamines in the homogenates were estimated. Data are expressed as mean ± standard error of the mean (SEM) for each group. Different symbols indicate significant differences at ^#^*p* < 0.05, ^##^*p* < 0.01, and ^###^*p* < 0.001 *vs.* normal group, and *p < 0.05, **p < 0.01, and ***p < 0.001 *vs.* control group. CPZ: chlorpromazine; L-DOPA: levodopa; 5-HTP: 5-hydroxytryptophan; ns: not significant.

The levels of depression-related neurotransmitters including epinephrine, L-DOPA, dopamine, 5-HTP, and serotonin, were analyzed by HPLC in the head of *Drosophila* (Figure 6). The epinephrine levels seemed to be lower in the control group than in the normal group, although the difference was not significant (Figure 6(A); *p* < 0.705). Dopamine (Figure 6(B); *p* < 0.003) and L-DOPA (Figure 6(C); *p* < 0.001) levels were significantly lower in the CPZ-treated control group than in the normal group. Conversely, there was a dose-dependent increase in L-DOPA and dopamine levels in the HE treatment groups (0.5, 1.0, and 1.5%) and significantly increased L-DOPA levels in the 1.5% HE-treated group compared to the normal group (Figures 6(B,C); *p* < 0.001). Meanwhile, 1.5% HE and CPZ-treated flies had significantly increased dopamine (1.41-fold; *p* < 0.036) and L-DOPA (1.38-fold; *p* < 0.001) levels compared to those in the control group (Figures 6(B,C)). Particularly, L-DOPA levels in 1.5% HE and CPZ-treated flies were similar to those in the flies of the normal group (Figure 6(C)). In addition, 5-HTP (Figure 6(D); *p* < 0.001) and serotonin (Figure 6(E); *p* < 0.040) levels were significantly decreased in the control group compared to the normal group. In contrast, flies administrated with 1.5% HE and CPZ showed significantly increased the 5-HTP (1.21-fold; *p* < 0.001) and serotonin (1.13-fold, *p* < 0.045) levels compared to the control group (Figures 6(D,E)). In only 1.5% HE-treated group, serotonin levels were significantly increased compared to the normal group (Figure 6(E); *p* < 0.016).

## Discussion

Depression is a multifaceted mental disorder, and symptoms of depression, such as loss of motivation and insensitivity to rewards, psychomotor changes, decreased sexual activity, and sleep or food intake disorders, can easily be quantified (Willner 1990). According to the monoamine hypothesis, deficiency of monoamine neuromodulators (serotonin, norepinephrine, and dopamine) in the central nervous system can lead to the development of depression (Manji et al. 2001). This study evaluated the anti-depressant effects of HE in a *D. melanogaster* model in which behavioural changes were induced by CPZ, which acts as a blocker of dopamine receptors.

*C. sativa* (hemp) seed, a by-product obtained during the commercial utilization of the plant fibre, has been used as a food and medicine in China for at least 3000 years (Callaway 2004). *C. sativa* also produces bioactive molecules called cannabinoids, the most unique and specific type of compounds known to exist only in cannabis plants. The active substances of HE are divided into psychoactive delta9-tetrahydrocannabinol (THC) and non-psychoactive CBD, a degradation product of THC (Messina et al. 2015). The ratio of THC + cannabinol/CBD is a criterion used to distinguish general hemp from medical hemp; if the ratio is <1 based on the overall ratio of 1, it is classified as industrial hemp, and if it exceeds 1, then it is classified as drug-type hemp (United Nations Office on Drug and Crime 2009). The content of cannabinoids in HE oil used for food is 1.36–12.40 mg/kg (Lachenmeier and Walch 2005). In general, the main cannabinoids present in fibrous plants are CBDA and its decarboxylated form CBD. CBD has anti-depressant and antioxidant activities, as well as anti-inflammatory, neuroprotective, anti-anxiety, and anticonvulsant properties (Appendino et al. 2011; Fernandez-Ruiz et al. 2011; de Mello Schier et al. 2014; Alexander 2016; Campos et al. 2016). Previous studies have shown that CBD at a dose of 30 mg/kg had a similar effect as imipramine, an anti-depressant (Reus et al. 2011). As a result of analysis by HR-MS, 32 cannabinoids were identified in 10 commercial hemp seeds. In this analysis, CBD and CBDA were abundant among these cannabinoids (Citti et al. 2019). The difference between the observed mass and the theoretical mass is used to measure the mass accuracy, and <2 ppm means high mass accuracy (Brenton and Godfrey 2010). As a result of HR-MS analysis, it was confirmed that HE contained cannabinoids, such as CBDA, CBDVA, CBDV, and THCV (Supplementary Figure 1 and Supplementary Table 1). Since the cannabinoids contents in HE is very low, it has a limitation to quantify it by HPLC. Therefore, analysis of the active substance should be carried out through further research.

*D. melanogaster* has shown depressive symptoms similar to those of humans when under stress (Shohat-Ophir et al. 2012; Yang et al. 2013). Additionally, L-DOPA and CPZ-treated *D. melanogaster* induce major behavioural and biochemical marker changes that are similar to depression (Jiang et al. 2017). CPZ is a potent antipsychotic drug used in the treatment of schizophrenia and other mental disorders and acts as a dopamine receptor antagonist (Harrold et al. 1987). Jiang et al. (2017) reported that CPZ induces depression in *D. melanogaster* as indicated by changes in appetite and sexual behaviour, increased malondialdehyde, decreased superoxide dismutase, and 5-HTP. Thus, 0.1% CPZ was used to induce depressive conditions in *D. melanogaster*. Figures 2, 3 demonstrate that the activity of CPZ-treated flies significantly decreased during the daytime and increased during the night-time compared to that of the normal group, and these changes in locomotor activity were similar to the symptoms of depression. Changes in night and day behaviour, or sleep disorders, are symptoms of depression (Vadnie and McClung 2017). During subjective daytime, the activity of HE and CPZ-treated flies increased compared to that of flies in the control group, and the activity of 1.5% HE and CPZ-treated flies was restored to approximately the baseline level. A previous study reported that vibrational stress-induced depression *Drosophila* showed changes in locomotor activities, such as decreased daytime activity and increased night-time activity. The reduction of daytime activities due to vibration stress was significantly increased by taurine treatment (0.05 and 0.10%) (Kim et al. 2020). Ries et al. (2017) reported that repeated vibration-stress in *Drosophila* reduced behavioural activity by inhibiting 5-HT release in the mushroom body. Administration of 5-HTP alleviates depression-like symptoms by preventing serotonin depletion in vibration-stressed *Drosophila*.

Depressive-like behavioural changes and neurochemical changes were measured to analyze the anti-depressant effects of HE. OFT is commonly used to assess the sedative, toxic, or stimulant effects of compounds. It is a general measure of exploratory behaviour and general activity in animals, and both quality and quantity of activity can be measured (Katz et al. 1981). In OFT, all factors of the movement were significantly reduced in flies in the control group compared to those in the normal group. The normal and 1.5% HE-treated groups showed similar behavioural patterns to distance moved, velocity, moving, and not moving (Figure 4). Compared to the results of previous studies, it was confirmed that the behavioural change according to the difference in sample concentration showed a similar trend to our results. Zanelati et al. (2010) reported that male Swiss mice treated high doses (30 mg/kg) of CBD were significantly increased immobility time in FST than the normal group. Conversely, mice treated with low doses (3 and 10 mg/kg) had increased immobility time than the normal group. In contrast, all factors of the movement were significantly increased in the 1.5% HE and CPZ-treated flies compared to the flies in the control group. The findings indicate that administration of 1.5% HE attenuated the depressant effect of CPZ. Animal behavioural models related to depression are evaluated for behavioural despair paradigms by FST and tail suspension test (TST) and stress paradigms, such as chronic mild stress, social stress, and early life stress (Krishnan and Nestler 2011). Mice exposed to chronic mild stress had increased immobility times in the FST and those exposed to chronic social defeat stress had decreased dopamine levels in the frontal cortex and raphe nuclei compared to mice in the control group (Venzala et al. 2013).

To assess the correlation between behavioural changes and mRNA expression, we used qRT-PCR analysis to identify levels of target genes *Dop1R1* and *5-HT1A* (Figure 5). Dopamine and serotonin receptor levels in the control group were significantly reduced compared to those in the normal group, whereas the levels of both receptor types significantly increased in the 1.5% HE and CPZ-treated group compared to the control group. Moraga-Amaro et al. (2014) reported that dopamine receptor subtype 3 deficiency causes depression-like symptoms as indicated by an increase in immobility in FST and TST of D3RKO mice. Moreover, depressed patients with anhedonia have significantly lower dopamine transporter binding than healthy subjects, as indicated in PET imaging studies (Sarchiapone et al. 2006).

Evidence of CPZ-induced depression was observed with neurotransmitter levels in the heads of flies and their inactivity time in the OFT. In the control group, dopamine, L-DOPA, 5-HTP, and serotonin levels were significantly decreased compared to those in the normal group (Figures 6(B–E)). However, administration of 1.5% HE improved catecholamine and serotonin levels compared to control. Dysfunction of the specific neurotransmitter system, including norepinephrine, dopamine, and serotonin, has been associated with pathological conditions of several neurological disorders. Specifically, dysfunction of dopamine signalling, which is the most abundant catecholamine in the mammalian brain, can cause problems with movement coordination, mood, memory, and emotions (Kobayashi 2001). For example, 1-methyl-4-phenyl-1,2,3,6-tetrahydropyridine (MPTP)-treated mice have prolonged immobility time in TST and decreased striatal dopamine levels. However, L-DOPA administration improves the MPTP-induced behavioural changes observed in TST and increases striatal dopamine levels (Mori et al. 2005).

We also investigated the effect of HE through changes in serotonin levels in a CPZ-induced depression model (Figure 6(E)). The deficiency of serotonin, which is related to anxiety, obsession, and compulsion, is also associated with depression (Fakhoury 2015). Araujo et al. (2021) reported that *Drosophila* exposed to unpredictable chronic mild stress (UCMS) had symptoms similar to depression due to decreased serotonin, dopamine, and octopamine. Administration of γ-oryzanol from rice bran oil have an effect on the improvement of *Drosophila* depression induced by UCMS through changes in molecular and genetic factors. In particular, γ-oryzanol treatment prevented the reduction of serotonin and octopamine in the depressed *Drosophila* model induced by UCMS. In addition, the development of antidepressants is related to theories based on serotonin deficiency, and numerous evidence suggests that various classes of antidepressant may induce enhancement of serotonin neurotransmission (Cowen and Browning 2015; Delcourte et al. 2021). Collectively, the results suggest that HE administration significantly ameliorates dopamine and serotonin levels.

## Conclusions

CPZ induces depression-like symptoms, such as changes in behavioural patterns, transcription levels of neurotransmitter receptors, and depression-related neurotransmitter levels in the *D. melanogaster* depression model. CPZ-treated flies have significantly reduced subjective daytime activity and increased immobility time. However, administration of HE restores the circadian rhythms, improves locomotor activity, and significantly increases transcription levels of dopamine and serotonin receptors in the depression-induced flies. Based on these findings, we can conclude that HE alleviates depression-like symptoms by increasing the levels of serotonin and dopamine receptors and dopamine, L-DOPA, 5-HTP, and serotonin levels in the brain. However, further studies are needed to investigate the role of HE administration in the depressive signalling pathways, changes in neurotransmitters, and behavioural patterns in vertebrate model systems.

## Supplementary Material

Supplemental Material

Supplemental Material

## References

[CIT0001] Alexander SPH. 2016. Therapeutic potential of cannabis-related drugs. Prog Neuropsychopharmacol Biol Psychiatry. 64:157–166. 26216862 10.1016/j.pnpbp.2015.07.001

[CIT0002] Anjaneyulu M, Chopra K, Kaur I. 2003. Antidepressant activity of quercetin, a bioflavonoid, in streptozotocin-induced diabetic mice. J Med Food. 6(4):391–395. 14977450 10.1089/109662003772519976

[CIT0003] Appendino G, Chianese G, Taglialatela-Scafati O. 2011. Cannabinoids: occurrence and medicinal chemistry. Curr Med Chem. 18(7):1085–1099. 21254969 10.2174/092986711794940888

[CIT0004] Araujo SM, Bortolotto VC, Poetini MR, Dahleh MMM, Couto SF, Pinheiro FC, Meichtry LB, Musachio EAS, Ramborger BP, Roehrs R, et al. 2021. γ-Oryzanol produces an antidepressant-like effect in a chronic unpredictable mild stress model of depression in *Drosophila melanogaster*. Stress. 24(3):282–212. 32723199 10.1080/10253890.2020.1790519

[CIT0005] Aryal B, Lee Y. 2019. Disease model organism for Parkinson disease: *Drosophila melanogaster*. BMB Rep. 52(4):250–258. 30545438 10.5483/BMBRep.2019.52.4.204 PMC6507844

[CIT0006] Bellen HJ, Tong C, Tsuda H. 2010. 100 years of *Drosophila* research and its impact on vertebrate neuroscience: a history lesson for the future. Nat Rev Neurosci. 11(7):514–522. 20383202 10.1038/nrn2839 PMC4022039

[CIT0007] Bolus H, Crocker K, Boekhoff-Falk G, Chtarbanova S. 2020. Modeling neurodegenerative disorders in *Drosophila melanogaster*. IJMS. 21(9):3055. 10.3390/ijms21093055 PMC7246467 32357532

[CIT0008] Brennenstuhl H, Jung-Klawitter S, Assmann B, Opladen T. 2019. Inherited disorders of neurotransmitters: classification and practical approaches for diagnosis and treatment. Neuropediatrics. 50(1):2–14. 30372766 10.1055/s-0038-1673630

[CIT0009] Brenton AG, Godfrey AR. 2010. Accurate mass measurement: terminology and treatment of data. J Am Soc Mass Spectrom. 21(11):1821–1835. 20650651 10.1016/j.jasms.2010.06.006

[CIT0010] Callaway J. 2004. Hempseed as a nutritional resource: an overview. Euphytica. 140(1–2):65–72.

[CIT0011] Campos AC, Fogaca MV, Sonego AB, Guimaraes FS. 2016. Cannabidiol, neuroprotection and neuropsychiatric disorders. Pharmacol Res. 112:119–127. 26845349 10.1016/j.phrs.2016.01.033

[CIT0012] Cassar M, Issa AR, Riemensperger T, Petitgas C, Rival T, Coulom H, Iché-Torres M, Han KA, Birman S. 2015. A dopamine receptor contributes to paraquat-induced neurotoxicity in *Drosophila*. Hum Mol Genet. 24(1):197–212. 25158689 10.1093/hmg/ddu430 PMC4326327

[CIT0013] Chandrasekhar Y, Ramya EM, Navya K, Phani Kumar G, Anilakumar KR. 2017. Antidepressant like effects of hydrolysable tannins of *Terminalia catappa* leaf extract via modulation of hippocampal plasticity and regulation of monoamine neurotransmitters subjected to chronic mild stress (CMS). Biomed Pharmacother. 86:414–425. 28012396 10.1016/j.biopha.2016.12.031

[CIT0014] Chia SJ, Tan EK, Chao YX. 2020. Historical perspective: models of Parkinson's disease. IJMS. 21(7):2464. 10.3390/ijms21072464 PMC7177377 32252301

[CIT0015] Citti C, Linciano P, Panseri S, Vezzalini F, Forni F, Vandelli MA, Cannazza G. 2019. Cannabinoid profiling of hemp seed oil by liquid chromatography coupled to high-resolution mass spectrometry. Front Plant Sci. 10:120. 30815007 10.3389/fpls.2019.00120 PMC6381057

[CIT0016] Cowen PJ, Browning M. 2015. What has serotonin to do with depression? World Psychiatry. 14(2):158–160. 26043325 10.1002/wps.20229 PMC4471964

[CIT0017] de Mello Schier AR, de Oliveira Ribeiro NP, Coutinho DS, Machado S, Arias-Carrion O, Crippa JA, Zuardi AW, Nardi AE, Silva AC. 2014. Antidepressant-like and anxiolytic-like effects of cannabidiol: a chemical compound of *Cannabis sativa*. CNS Neurol Disord Drug Targets. 13(6):953–960. 24923339 10.2174/1871527313666140612114838

[CIT0018] Dean J, Keshavan M. 2017. The neurobiology of depression: an integrated view. Asian J Psychiatr. 27:101–111. 28558878 10.1016/j.ajp.2017.01.025

[CIT0019] Delcourte S, Etievant A, Haddjeri N. 2021. Role of central serotonin and noradrenaline interactions in the antidepressants' action: electrophysiological and neurochemical evidence. Prog Brain Res. 259:7–81. 33541681 10.1016/bs.pbr.2021.01.002

[CIT0020] Denno ME, Privman E, Venton BJ. 2015. Analysis of neurotransmitter tissue content of *Drosophila melanogaster* in different life stages. ACS Chem Neurosci. 6(1):117–123. 25437353 10.1021/cn500261e PMC4304510

[CIT0021] Dunlop BW, Nemeroff CB. 2007. The role of dopamine in the pathophysiology of depression. Arch Gen Psychiatry. 64(3):327–337. 17339521 10.1001/archpsyc.64.3.327

[CIT0022] El-Alfy AT, Ivey K, Robinson K, Ahmed S, Radwan M, Slade D, Khan I, ElSohly M, Ross S. 2010. Antidepressant-like effect of delta9-tetrahydrocannabinol and other cannabinoids isolated from *Cannabis sativa* L. Pharmacol Biochem Behav. 95(4):434–442. 20332000 10.1016/j.pbb.2010.03.004 PMC2866040

[CIT0023] Fakhoury M. 2015. New insights into the neurobiological mechanisms of major depressive disorders. Gen Hosp Psychiatry. 37(2):172–177. 25772946 10.1016/j.genhosppsych.2015.01.005

[CIT0024] Fernandez-Ruiz J, Moreno-Martet M, Rodriguez-Cueto C, Palomo-Garo C, Gomez-Canas M, Valdeolivas S, Guaza C, Romero J, Guzman M, Mechoulam R, et al. 2011. Prospects for cannabinoid therapies in basal ganglia disorders. Br J Pharmacol. 163(7):1365–1378. 21545415 10.1111/j.1476-5381.2011.01365.x PMC3165947

[CIT0025] Harrold MW, Chang YA, Wallace RA, Farooqui T, Wallace LJ, Uretsky N, Miller DD. 1987. Charged analogues of chlorpromazine as dopamine antagonists. J Med Chem. 30(9):1631–1635. 2887659 10.1021/jm00392a019

[CIT0026] Hendricks JC, Finn SM, Panckeri KA, Chavkin J, Williams JA, Sehgal A, Pack AI. 2000. Rest in *Drosophila* is a sleep-like state. Neuron. 25(1):129–138. 10707978 10.1016/s0896-6273(00)80877-6

[CIT1111] Hood S, Amir S. 2017. Neurodegeneration and the circadian clock. Front Aging Neurosci. 9:170. 10.3389/fnagi.2017.00170 PMC5447688 28611660

[CIT0027] Jiang MD, Zheng Y, Wang JL, Wang YF. 2017. Drug induces depression-like phenotypes and alters gene expression profiles in *Drosophila*. Brain Res Bull. 132:222–231. 28625786 10.1016/j.brainresbull.2017.06.009

[CIT0028] Katz RJ, Roth KA, Carroll BJ. 1981. Acute and chronic stress effects on open field activity in the rat: implications for a model of depression. Neurosci Biobehav Rev. 5(2):247–251. 7196554 10.1016/0149-7634(81)90005-1

[CIT0029] Kaur K, Simon AF, Chauhan V, Chauhan A. 2015. Effect of bisphenol A on *Drosophila melanogaster* behavior-a new model for the studies on neurodevelopmental disorders. Behav Brain Res. 284:77–84. 25660202 10.1016/j.bbr.2015.02.001

[CIT0030] Keles B, McCrae N, Grealish A. 2020. A systematic review: the influence of social media on depression, anxiety and psychological distress in adolescents. Int J Adolesc Youth. 25(1):79–93.

[CIT0031] Kim S, Hong KB, Kim S, Suh HJ, Jo K. 2020. Creatine and taurine mixtures alleviate depressive-like behaviour in *Drosophila melanogaster* and mice via regulating Akt and ERK/BDNF pathways. Sci Rep. 10(1):11370. 32647316 10.1038/s41598-020-68424-1 PMC7347602

[CIT0032] Ko CH, Koon CM, Yu SL, Lee KY, Lau CBS, Chan EHY, Wing YK, Fung KP, Leung PC. 2016. Hypnotic effects of a novel anti-insomnia formula on *Drosophila* insomnia model. Chin J Integr Med. 22(5):335–343. 25159862 10.1007/s11655-014-1625-1

[CIT0033] Kobayashi K. 2001. Role of catecholamine signaling in brain and nervous system functions: new insights from mouse molecular genetic study. J Investig Dermatol Symp Proc. 6(1):115–121. 10.1046/j.0022-202x.2001.00011.x 11764279

[CIT0034] Krishnan V, Nestler EJ. 2011. Animal models of depression: molecular perspectives. Curr Top Behav Neurosci. 7:121–147. 21225412 10.1007/7854_2010_108 PMC3270071

[CIT0035] Kulkarni SK, Bhutani MK, Bishnoi M. 2008. Antidepressant activity of curcumin: involvement of serotonin and dopamine system. Psychopharmacology. 201(3):435–442. 18766332 10.1007/s00213-008-1300-y

[CIT0036] Kumar A, Sharma B, 2020. Phytochemicals as antidepressants. In: Patra JK, Shukla AC, Das G, editors. Advances in pharmaceutical biotechnology. New York (NY): Springer; p. 115–131.

[CIT0037] Kurkin V, Dubishchev A, Ezhkov V, Titova I, Avdeeva E. 2006. Antidepressant activity of some phytopharmaceuticals and phenylpropanoids. Pharm Chem J. 40(11):614–619.

[CIT0038] Kwon S, Lee B, Kim M, Lee H, Park HJ, Hahm DH. 2010. Antidepressant-like effect of the methanolic extract from *Bupleurum falcatum* in the tail suspension test. Prog Neuropsychopharmacol Biol Psychiatry. 34(2):265–270. 19932727 10.1016/j.pnpbp.2009.11.015

[CIT0039] Lachenmeier DW, Walch SG. 2005. Analysis and toxicological evaluation of cannabinoids in hemp food products–a review. Elec J Env Agric Food Chem. 4:812–826.

[CIT0040] Lavergne F, Jay TM. 2010. A new strategy for antidepressant prescription. Front Neurosci. 4:192. 21151361 10.3389/fnins.2010.00192 PMC2995552

[CIT0041] Lee SH, Min KJ. 2015. Life-extending effect of phytochemicals in *Drosophila*. In: Vaiserman AM, Moskalev AA, Pasyukova EG, editors. Life extension. New York (NY): Springer; p. 229–244.

[CIT0042] Ligresti A, De Petrocellis L, Di Marzo V. 2016. From phytocannabinoids to cannabinoid receptors and endocannabinoids: pleiotropic physiological and pathological roles through complex pharmacology. Physiol Rev. 96(4):1593–1659. 27630175 10.1152/physrev.00002.2016

[CIT0043] Livak KJ, Schmittgen TD. 2001. Analysis of relative gene expression data using real-time quantitative PCR and the 2(-Delta Delta C(T)) method. Methods. 25(4):402–408. 11846609 10.1006/meth.2001.1262

[CIT0044] Mandrioli M, Tura M, Scotti S, Toschi TG. 2019. Fast detection of 10 cannabinoids by RP-HPLC-UV method in *Cannabis sativa L*. Molecules. 24(11):2113. 10.3390/molecules24112113 PMC6600594 31167395

[CIT0045] Manji HK, Drevets WC, Charney DS. 2001. The cellular neurobiology of depression. Nat Med. 7(5):541–547. 11329053 10.1038/87865

[CIT0046] Martin CA, Krantz DE. 2014. *Drosophila melanogaster* as a genetic model system to study neurotransmitter transporters. Neurochem Int. 73:71–88. 24704795 10.1016/j.neuint.2014.03.015 PMC4264877

[CIT0047] Messina F, Rosati O, Curini M, Marcotullio MC. 2015. *Cannabis* and bioactive cannabinoids: Studies in natural products chemistry. In: Atta U, editor. Studies in natural products chemistry. Oxford: Elsevier; p. 17–57.

[CIT0048] Mori A, Ohashi S, Nakai M, Moriizumi T, Mitsumoto Y. 2005. Neural mechanisms underlying motor dysfunction as detected by the tail suspension test in MPTP-treated C57BL/6 mice. Neurosci Res. 51(3):265–274. 15710490 10.1016/j.neures.2004.11.008

[CIT2222] Moraga-Amaro R, Gonzalez H, Pacheco R, Stehberg J. 2014. Dopamine receptor D3 deficiency results in chronic depression and anxiety. Behav Brain Res. 274:186–193. 10.1016/j.bbr.2014.07.055 25110304

[CIT0049] Nathan PJ. 2001. *Hypericum perforatum* (St John's Wort): a non-selective reuptake inhibitor? A review of the recent advances in its pharmacology. J Psychopharmacol. 15(1):47–54. 11277608 10.1177/026988110101500109

[CIT0050] Noldner M, Schotz K. 2002. Rutin is essential for the antidepressant activity of *Hypericum perforatum* extracts in the forced swimming test. Planta Med. 68:577–580. 12142988 10.1055/s-2002-32908

[CIT0051] O'Kane CJ. 2011. *Drosophila* as a model organism for the study of neuropsychiatric disorders. Curr Top Behav Neurosci. 7:37–60. 21225410 10.1007/7854_2010_110

[CIT0052] Pathak L, Agrawal Y, Dhir A. 2013. Natural polyphenols in the management of major depression. Expert Opin Investig Drugs. 22(7):863–880. 10.1517/13543784.2013.794783 23642183

[CIT0053] Resstel LB, Joca SR, Moreira FA, Correa FM, Guimaraes FS. 2006. Effects of cannabidiol and diazepam on behavioral and cardiovascular responses induced by contextual conditioned fear in rats. Behav Brain Res. 172(2):294–298. 16780966 10.1016/j.bbr.2006.05.016

[CIT0054] Reus GZ, Stringari RB, Ribeiro KF, Luft T, Abelaira HM, Fries GR, Aguiar BW, Kapczinski F, Hallak JE, Zuardi AW, et al. 2011. Administration of cannabidiol and imipramine induces antidepressant-like effects in the forced swimming test and increases brain-derived neurotrophic factor levels in the rat amygdala. Acta Neuropsychiatr. 23(5):241–248. 25379896 10.1111/j.1601-5215.2011.00579.x

[CIT0055] Ries AS, Hermanns T, Poeck B, Strauss R. 2017. Serotonin modulates a depression-like state in *Drosophila* responsive to lithium treatment. Nat Commun. 8(1):1–11. 28585544 10.1038/ncomms15738 PMC5467214

[CIT0056] Russo EB. 2007. History of cannabis and its preparations in saga, science, and sobriquet. Chem Biodivers. 4(8):1614–1648. 17712811 10.1002/cbdv.200790144

[CIT0057] Sarchiapone M, Carli V, Camardese G, Cuomo C, Di Giuda D, Calcagni ML, Focacci C, De Risio S. 2006. Dopamine transporter binding in depressed patients with anhedonia. Psychiatry Res. 147(2–3):243–248. 16952448 10.1016/j.pscychresns.2006.03.001

[CIT0058] Shohat-Ophir G, Kaun KR, Azanchi R, Mohammed H, Heberlein U. 2012. Sexual deprivation increases ethanol intake in *Drosophila*. Science. 335(6074):1351–1355. 22422983 10.1126/science.1215932 PMC3909676

[CIT0059] Shukla AK, Pragya P, Chaouhan HS, Patel DK, Abdin MZ, Kar Chowdhuri D. 2014. Mutation in *Drosophila* methuselah resists paraquat induced Parkinson-like phenotypes. Neurobiol Aging. 35(10):2419.e1–2419.e6. 10.1016/j.neurobiolaging.2014.04.008 24819147

[CIT0060] Silote GP, Sartim A, Sales A, Eskelund A, Guimarães FS, Wegener G, Joca S. 2019. Emerging evidence for the antidepressant effect of cannabidiol and the underlying molecular mechanisms. J Chem Neuroanat. 98:104–116. 31039391 10.1016/j.jchemneu.2019.04.006

[CIT0061] Thanacoody R. 2020. Antidepressant and antipsychotic poisoning. Medicine. 48(3):194–196.

[CIT0062] United Nations Office on Drug and Crime. 2009. Recommended methods for the identification and analysis of Cannabis and Cannabis products: manual for use by national drug testing laboratories. New York (NY): United Nations Publications; p. 1–50.

[CIT0063] Vadnie CA, McClung CA. 2017. Circadian rhythm disturbances in mood disorders: insights into the role of the suprachiasmatic nucleus. Neural Plast. 2017:1504507. 29230328 10.1155/2017/1504507 PMC5694588

[CIT0064] Venzala E, Garcia-Garcia AL, Elizalde N, Tordera RM. 2013. Social vs. environmental stress models of depression from a behavioural and neurochemical approach. Eur Neuropsychopharmacol. 23(7):697–708. 22743048 10.1016/j.euroneuro.2012.05.010

[CIT0065] Weston-Green K. 2018. The united chemicals of cannabis: Beneficial effects of cannabis phytochemicals on the brain and cognition. In: Costain W, Lapraire RB, editors. Recent advances in cannabinoid research. Ukraine: InTechOpen; p. 1–19.

[CIT0066] Willner P. 1990. Animal models of depression: an overview. Pharmacol Therapeut. 45(3):425–455. 10.1016/0163-7258(90)90076-e 2405444

[CIT0067] Xu Y, Li S, Chen R, Li G, Barish PA, You W, Chen L, Lin M, Ku B, Pan J, et al. 2010. Antidepressant-like effect of low molecular proanthocyanidin in mice: involvement of monoaminergic system. Pharmacol Biochem Behav. 94(3):447–453. 19857512 10.1016/j.pbb.2009.10.007

[CIT0068] Yang Z, Bertolucci F, Wolf R, Heisenberg M. 2013. Flies cope with uncontrollable stress by learned helplessness. Curr Biol. 23(9):799–803. 23602474 10.1016/j.cub.2013.03.054

[CIT3333] Zanelati TV, Biojone C, Moreira FA, Guimarães FS, Joca SR. 2010. Antidepressant-like effects of cannabidiol in mice: possible involvement of 5-HT1A receptors. Br J Pharmacol. 159(1):122–128. 10.1111/j.1476-5381.2009.00521.x PMC2823358 20002102

[CIT0069] Zhu X, Li W, Li Y, Xu W, Yuan Y, Zheng V, Zhang H, O'Donnell JM, Xu Y, Yin X. 2019. The antidepressant- and anxiolytic-like effects of resveratrol: involvement of phosphodiesterase-4D inhibition. Neuropharmacology. 153:20–31. 31026437 10.1016/j.neuropharm.2019.04.022

[CIT0070] Zuardi AW, Crippa JA, Hallak JE, Moreira FA, Guimaraes FS. 2006. Cannabidiol, a *Cannabis sativa* constituent, as an antipsychotic drug. Braz J Med Biol Res. 39(4):421–429. 16612464 10.1590/s0100-879x2006000400001

[CIT0071] Zuardi AW, Shirakawa I, Finkelfarb E, Karniol IG. 1982. Action of cannabidiol on the anxiety and other effects produced by delta 9-THC in normal subjects. Psychopharmacology. 76(3):245–250. 6285406 10.1007/BF00432554

