# Supplementary figures and images for: Anti-depressant effects of ethanol extract from Cannabis sativa (hemp) seed in chlorpromazine-induced Drosophila melanogaster depression model
Source: Pharm Biol. 2021 Aug 6;59(1):996–1005. doi: 10.1080/13880209.2021.1949356 (PMC8354181; doi:10.1080/13880209.2021.1949356)

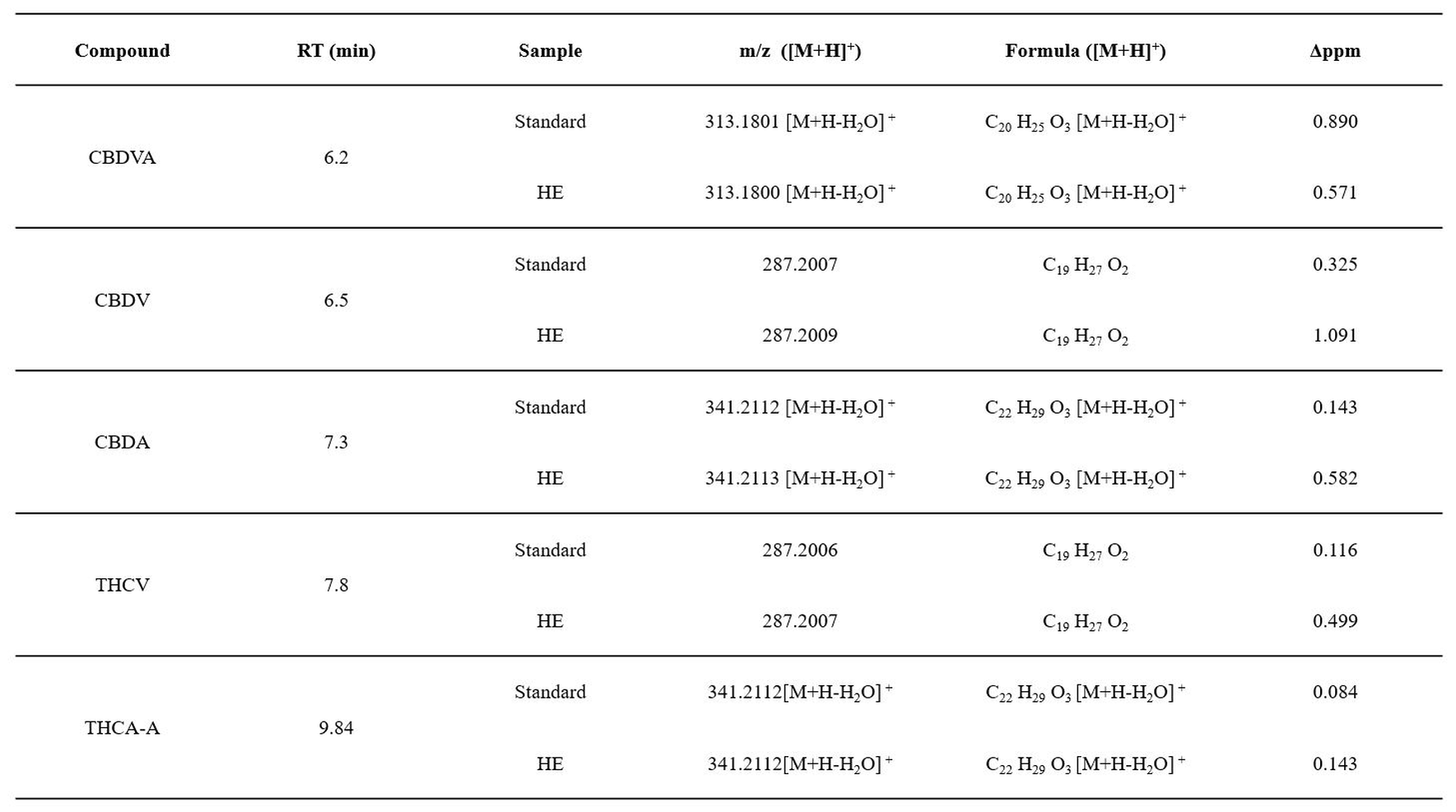

Supplement: Supplemental Material [file IPHB_A_1949356_SM2290.tif]

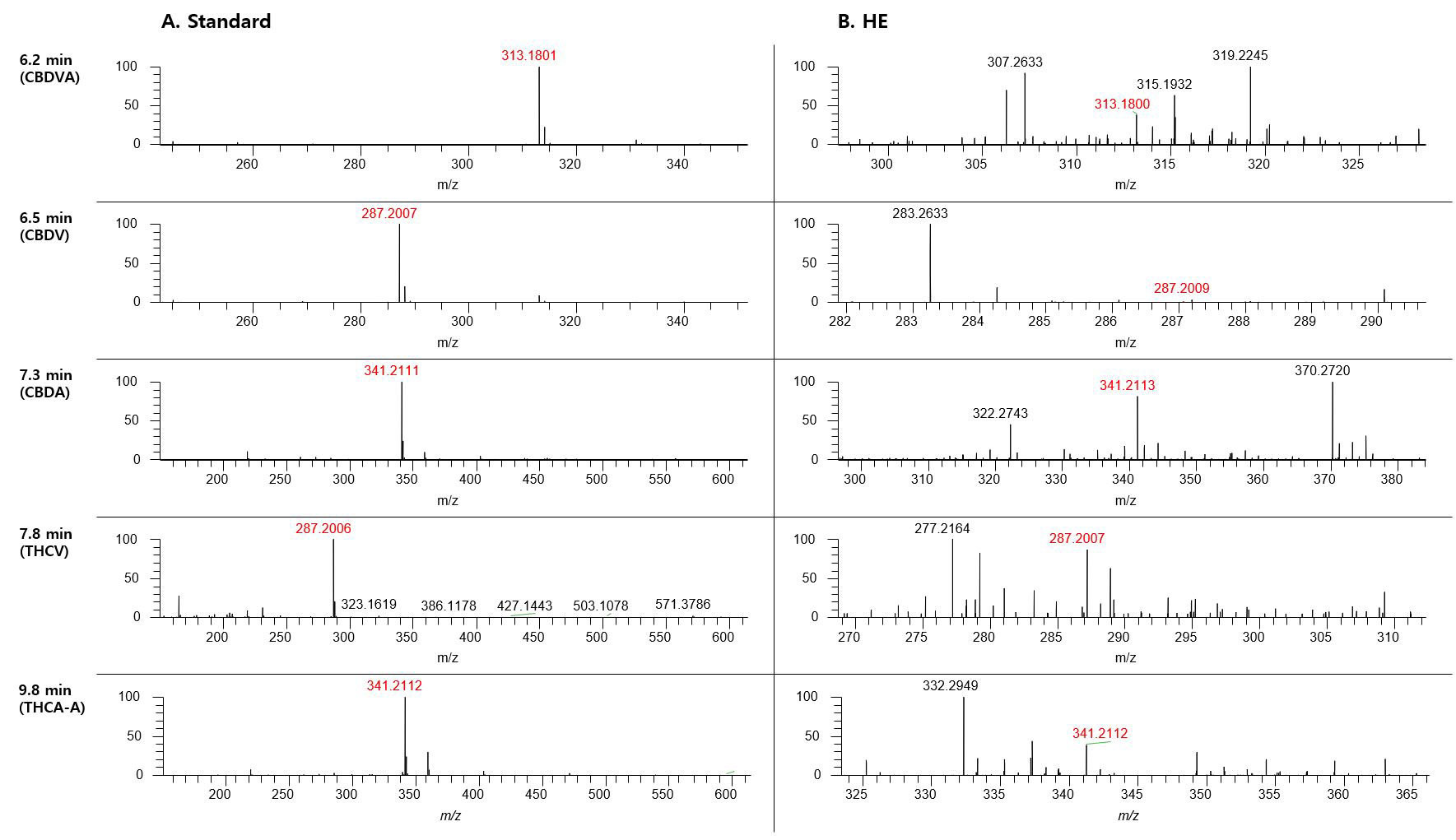

Supplement: Supplemental Material [file IPHB_A_1949356_SM2288.tif]
